# Effects of Aqueous Extract of Fresh Leaves of *Abroma augusta* L. on Oral Absorption of Glucose and Metformin Hydrochloride in Experimental Rats

**DOI:** 10.5402/2012/472586

**Published:** 2012-07-05

**Authors:** Tariqul Islam, Ajijur Rahman, Anwar Ul Islam

**Affiliations:** Department of Pharmacy, University of Rajshahi, Rajshahi 6205, Bangladesh

## Abstract

To get better control in disease conditions, many people take herbs with conventional medicines, therefore, posing a risk of potential pharmacokinetic interactions between herbs and conventional drugs. The aqueous extract of the fresh leaves of *Abroma augusta* L. (Family: Sterculiaceae, Bengali name: Ulatkambal, English name: Devil's cotton, DC) is viscous and used traditionally to treat diabetes mellitus. This study was done to investigate the probable mechanism by which the aqueous extract of *Abroma augusta* L. is beneficial in managing type 2 DM and to observe the effects of this extract on absorption of metformin hydrochloride from the gastrointestinal tract. Studies were conducted in healthy Long Evans rats using Na-carboxymethyl cellulose (CMC) as positive control. Both Na-CMC and WSF of DC significantly (*P* < 0.05) reduced the absorption of glucose administered orally in fasted rats. On the other hand, WSF of DC significantly (*P* < 0.05) reduced the absorption of metformin hydrochloride in alloxan-induced diabetic rats. The results of this study suggest that WSF of DC may be beneficial in diabetic patients to improve glycemic control but should not be coadministered with metformin HCl for management of type 2 diabetes mellitus.

## 1. Introduction

Traditional herbal medicines have been attracting a great deal of attention as alternative and supplemental medicines. They are frequently prescribed with western drugs for the treatment of chronic diseases in many developed countries like Japan [1]. In someAsian andAfrican countries, up to 80% of the population relies on traditional medicine for theirprimary health care needs [2]. WHO also states that traditional medicines are not as always safe as many people believe; they may cause harmful, adverse reactions if the product or therapy is of poor quality, or it is taken inappropriately or in conjunction with other medicines. Potential herb-drug interactions occur with drugs metabolized by CYP1 and CYP2 enzymes. Garlic (*Allium sativum*), for instance, inhibits CYP enzyme; thus the metabolism of several drugs is reduced causing toxicity [3]. Physical interaction of herbs and drugs also results in reduced and delayed absorption. 

Recently, we have reported that the viscous WSF of *Abelmoschus esculentus *L. (lady's fingers) interacts with both glucose and metformin hydrochloride and reduces their absorption from gastrointestinal tract when co-administered orally in rats. From these observations we suggested that although lady's fingers are beneficial in type 2 DM patients in improving glucose tolerance, they should not be taken with metformin at the same time [4]. The aqueous extract of fresh leaves of *A. augusta *L. is highly viscous, almost similar to the aqueous extract of the green pods of *Abelmoschus esculentus *L. Both of them are used ethnomedicinally to treat type 2 DM [5]. 

The antidiabetic activity of aqueous extract of dried root bark of *Abroma augusta* L. has been observed in diabetic rats by Halim [6] and Ali Hussain [7]. They showed that the water extract of the roots of *A. augusta* L. resulted in significant fall in fasting blood glucose and improvement in glucose tolerance. Nahar et al. reported that the methanolic extract of leaves of *Abroma augusta* L. significantly reduced the blood glucose level in alloxan-induced diabetic rats when administered at a dose of 300 mg/kg/day [8]. These findings established the rationality of ethnomedicinal use of the roots and leaves of DC in management of diabetes.

Many studies have shown that many people take herbs with conventional medicines without informing their physicians. They are unaware of the possible pharmacokinetic interactions of these herbal drugs with prescribed western drugs. Therefore, in this study, we examined the effects of simultaneous oral administration of aqueous leaf extract of *A. augusta* L. and metformin on the absorption of metformin from gastrointestinal tract in diabetic rats. We also examined how aqueous leaf extract improves glycemic control by reducing absorption of glucose in fasting rats.

## 2. Materials and Methods

### 2.1. Materials

Metformin hydrochloride was obtained as a gift sample from Square Pharmaceuticals Limited, Dhaka, Bangladesh. Na-CMC (Fluka, Switzerland, pH = 6.5–8.0; 400–1000 mPa.s), glucose (dextrose monohydrate, GSK), alloxan monohydrate (Loba Chemicals, India), saline water (Saloride, Beximco Pharmaceuticals), insulin syringe (100 units), gastric feeding tube, glucose test strip, and glucose meter (Bioland, Germany) were purchased from local suppliers.

### 2.2. Collection of Fresh Leaves and Preparation of Viscous Water-Soluble Fractions

Fresh leaves of *Abroma augusta* L. were collected in the month of October, 2010, from Botanical Garden, Rajshahi University, Rajshahi, Bangladesh. After collection, the fresh leaves were thoroughly washed with distilled water and sliced into small pieces. The sliced leaves (60 gm) were immersed into 250 mL distilled water in a beaker and stirred gently for 10 to 15 minutes with a glass rod. After 24 hours, the mixture was filtered and the viscous WSF was collected as a filtrate. Finally, the viscosity was measured using Brookfield Dial Viscometer (LV series, Brookfield Engineering Laboratories, Inc., USA). 

### 2.3. Maintenance of Experimental Animals

To perform these experiments, 30 healthy Long Evans rats (six weeks old, weighing about 100–150 gm) were collected from ICDDRB, Bangladesh. They were maintained in colony cages (3 rats per cage) at an ambient temperature of 25–27°C with 12-hour light and dark cycles having proper ventilation in the room and were fed normal diets and water. They were allowed one week to acclimatize to the laboratory environment and were randomly divided into groups for experiments.

### 2.4. Induction of Diabetes in Rats by Alloxan

Rats were allowed to fast for 12 hrs, and freshly prepared solution of alloxan (120 mg/kg b. w.) in normal saline water was administered intraperitoneally with an insulin syringe (100 units). After treatments, rats were allowed to feed normal diet. After 48 hours, glucose level was measured in the blood collected by pricking tail veins. Rats that were not diabetic (<14.7 mmol/L) or that were extremely diabetic (>35.5 mmol/L) were excluded from the study.

### 2.5. Preparation of Different Dosing Mixtures

#### 2.5.1. Glucose Solution

Glucose solution was prepared by dissolving glucose in distilled water in such a concentration that each 0.3 mL of solution contains 1.20 gm/kg body weight of glucose. 

#### 2.5.2. Glucose-CMC

0.3 mL of glucose solution was mixed with 0.2 mL of Na-CMC (4 mg/mL).

#### 2.5.3. Glucose-WSF of *Abroma augusta* L.

0.3 mL of glucose solution was mixed with 0.5 mL of WSFUK (19 mg/mL).

#### 2.5.4. Metformin HCl

Metformin HCl solution was prepared by dissolving metformin in sterile normal saline water in such a concentration that each 0.2 mL of solution contains metformin hydrochloride with a single dose of 500 mg/kg body weight of alloxan-induced diabetic rats. 

#### 2.5.5. Metformin HCl-CMC

0.2 mL of metformin HCl solution was mixed with 0.2 mL of Na-CMC (8 mg/mL).

#### 2.5.6. Metformin HCl-WSF of *Abroma augusta* L.

0.2 mL of metformin HCl solution was mixed with 0.5 mL of WSFUK (19 mg/mL).

### 2.6. Experimental Design and Administration of Doses

To investigate the effects of water-soluble fraction of fresh leaves of *A. augusta* L. on glucose absorption from gastrointestinal tract, 15 rats were divided into the following five groups (*n* = 3 per group): normal control (NC), fasting control (FC), fasting with glucose only (glucose control, FG), fasting with glucose and CMC (FGC), and fasting with glucose and WSF of *A. augusta *L. (FGF). Rats of all groups except normal control group were deprived of diet and water for 24 hours to eliminate the relevant gastrointestinal factors. After 24 hours fasting blood glucose levels were measured. Then, 0.3 mL glucose (1.20 gm/kg b. w.), dosing mixture of glucose-Na-CMC (0.3 mL glucose with 0.2 mL), and dosing mixture of glucose-WSFUK (0.3 mL glucose with 0.5 mL WSF) were administered orally to the respective groups of rats by means of a gastric feeding tube. The fasting control rats were fed only sterile distilled water whereas normal control rats were fed normal diet and sterile distilled water.

To observe the effects of WSF of *A. augusta* L. on the oral absorption of metformin hydrochloride, 15 healthy Long Evans rats were randomly divided into five groups (*n* = 3 per group): normal control (NC), diabetic control (DC), diabetic with metformin hydrochloride only (metformin control, MC), diabetic with metformin hydrochloride and CMC (DMC), and diabetic with metformin hydrochloride and WSF of *A. augusta* L. (DMF). Metformin HCl was dissolved in distilled water. 0.2 mL of metformin (500 mg/kg b. w.), dosing mixtures of metformin-CMC (0.2 mL metformin with 0.2 mL CMC), and dosing mixtures of metformin-WSFUK (0.2 mL metformin with 0.5 mL WSF of *A. augusta* L.) were orally administered to the alloxan-induced diabetic rats by a gastric feeding tube. The diabetic control rats were fed only distilled water whereas normal control rats were fed normal diet and distilled water. After the treatment blood, glucose level was estimated at different time intervals.

### 2.7. Statistical Analysis

All results were expressed as mean ± SD of three replicates. All data were analyzed with two-way analysis of variance (ANOVA) followed by Dunnett's test using statistical software SPSS 15.0. The level of significance was set at *P* < 0.05. The figures were prepared using MS-Excel 2003.

## 3. Results and Discussions

### 3.1. Effect of WSF of *A. augusta* L. on Glucose Absorption in 24-Hour Fasted Rats

**Figure 1:**
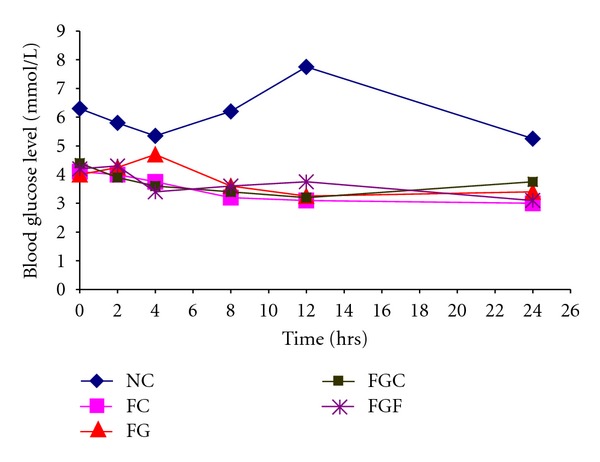
Effects of orally administered Na-CMC and WSF of *A. augusta* L. on glucose absorption in 24-hour fasted rats. NC: normal control; FC: fasting control; FG: fasting with glucose (1.2 gm/kg b. w.); FGC: fasting with glucose (1.2 gm/kg b. w.) and CMC; FGF: fasting with glucose (1.2 gm/kg b. w.) and WSF of *A. augusta *L.

As shown in Figure 1 and Table 1, WSF of *A. augusta* L. reduces the absorption of glucose in 24-hour fasted rats. In the FG group of rats, we have seen 17.5% increases in blood glucose level at 4 hrs after administration of glucose (from initial 4.0 mmol/L to 4.70 mmol/L). On the other hand, when same amount of glucose was administered with 0.5 mL WSF of *A. augusta *L., blood glucose level rather decreased to about 49.26% at 4 hrs (from initial 6.70 mmol/L to 3.40 mmol/L), and the same trend continued afterwards. The inhibitory effects of *A. augusta* L. were much more higher than CMC. 

We assume that this type of inhibition of metformin activity by the aqueous fraction of leafs of *Abroma augusta* L. may be due to the entrapment of glucose by the viscous soluble fibers present in the extract. Presence of water-soluble fibers like gum and pectin in the aqueous extract may be the reason. To prove whether the inhibition is due to interaction of fiber and drug or other factors like pharmacokinetic interaction are involved, we designed and performed an *in vitro* study. We observed that the aqueous fraction of *Abroma augusta* L. significantly hamper the diffusion of glucose through ultrafine membrane into the outer solution (data not shown). Previous studies also reported that viscous water-soluble fibers hamper the diffusion of glucose and postpone the absorption and digestion of carbohydrates resulting in lowered postprandial blood glucose level [9–12]. Soluble dietary fiber such as guar gum and pectin has been shown to lower blood glucose level and insulin in human, when given simultaneously with a carbohydrate-containing meal [13]. Dietary fibers lower the glycemic level in serum by slowing glucose absorption through an effect on gastric emptying and/or entrapment of materials in the viscous digesta [14]. Schwartz and Levine, found that chronic fiber supplementation significantly decreases intestinal glucose absorption in rats [15]. It has also been observed that different types of dietary fibers (especially soluble) reduced the diffusion of glucose both* in vitro* and *in vivo* [16, 17]. Our findings along with others may conclude that probably the fibers contributing to the viscosity of the water-soluble fraction of this plant inhibit the absorption of glucose from the gastrointestinal tract of rats by entrapment and thus are beneficial to the type 2 DM patients to improve glucose tolerance.

### 3.2. Effect of WSF of *Abroma augusta* L. on Metformin Absorption in Alloxan-Induced Diabetic Rats

**Figure 2:**
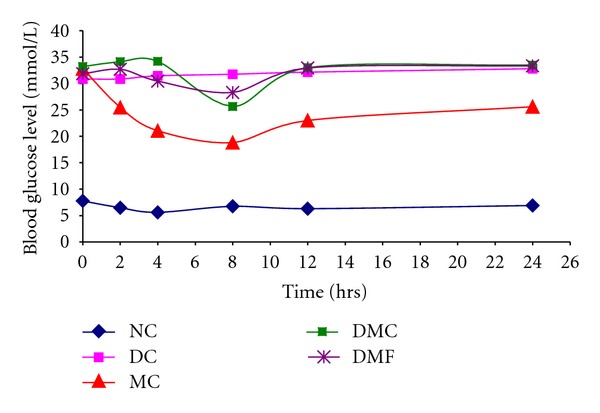
Effects of orally administered CMC and WSF of *A. augusta* L. on metformin HCl absorption in alloxan-induced diabetic rats. NC: normal control; DC: diabetic control; MC: diabetic with metformin only (metformin control); DMC: diabetic with metformin and CMC; DMF: diabetic with metformin and WSF of *A. augusta* L.

The effect of WSF of *A. augusta* L. on the absorption of metformin hydrochloride was examined in alloxan-induced diabetic rats. We observed that the WSF of fresh leaves of *A. augusta* L. significantly reduced the absorption of metformin hydrochloride which results in nearly complete loss of antihyperglycemic effect of metformin (Table 2, Figure 2). The initial average blood glucose level in metformin control rats (metformin HCl was administered without WSF of *A. augusta *L.) decreased to about 44.8% within 8 hours (from initial 32.80 mmol/L to 18.80 mmol/L).

In contrast, coadministration of metformin HCl and WSF of ulatkambal in DMF group of rats, blood glucose level decreased to only 11.14% from the initial level on 8 hrs (from initial 31.85 mmol/L to 28.30 mmol/L). Moreover, it is clear from Figure 2 that both CMC and WSF of devil's cotton inhibited activities of metformin till 4 hrs after administration and during this time blood glucose level has been changed significantly. After that time a little amount is released which is reflected in the glucose level. This experimental result can be explained by the fact that WSF of *Abroma augusta* L. and CMC entrapped metformin and significantly inhibited its absorption. The molecular interaction between the amine group of metformin and carboxyl groups of the fiber components of WSF may be the reason of this entrapment [4]. High viscosity of WSF of extract of leaves (30 cP) may also contribute to this effect by inhibiting the diffusion of metformin from the dosing mixtures. In a study, Iwao and his coworkers have observed that metformin hydrochloride interacts differently with three healthy foods aojiru, kurosu, and blueberry extract and modulate their absorption [16]. 

## 4. Conclusion

This simple investigation proved that the aqueous extract of fresh leaves of *Abroma augusta* L. which is used by many people as an alternative treatment for type 2 DM reduces the absorption of glucose, thus helps in glucose tolerance. At the same time we have also shown that probably the water-soluble fibers present in the aqueous fraction also substantially reduced metformin activity in diabetic rats. We assume that this inhibition is due to physical and/or chemical interaction of metformin and the water-soluble fibers of the extract. But the exact mechanism by which the WSF of *Abroma augusta* L. inhibits the absorption of co-administered drugs is needed to be investigated.

## Figures and Tables

**Table 1:** Blood glucose level (mmol/L) after oral administration of glucose, WSF of *A. augusta* L. and CMC in rats kept for fasting for 24 hrs.

Blood glucose level (mmol/L)						
Treatment groups	0 hr	2 hrs	4 hrs	8 hrs	12 hrs	24 hrs
NC	6.30 ± 0.42	5.80 ± 0.28	5.35 ± 0.92	6.20 ± 0.85	7.75 ± 3.18	5.25 ± 0.35
FC	4.10 ± 0.14	4.0 ± 0	3.75 ± 0.21	3.20 ± 0.28	3.10 ± 0.14	3.0 ± 0
FG	4.0 ± 0	4.25 ± 0.07	4.70 ± 0.14	3.60 ± 0.14	3.25 ± 0.07	3.40 ± 0.28
FGC	4.40 ± 0.71	3.90 ± 0.28	3.60 ± 0.57^a^	3.40 ± 0.42	3.20 ± 0.14	3.75 ± 0.07
FGF	6.70 ± 1.27	4.30 ± 0	3.40 ± 0.57^a^	3.60 ± 0.42	3.75 ± 0.64	3.10 ± 0.14

Values are expressed as mean ± SD of three replicates. NC: normal control; FC: fasting control; FG: fasting with glucose (1.2 gm/kg b. w.); FGC: fasting with glucose and CMC; FGF: fasting with glucose (1.2 gm/kg b. w.); WSF of *A. augusta *L. ^a^ *P* < 0.05 compared to FG (ANOVA followed by Dunnett's test).

**Table 2:** Blood glucose level (mmol/L) in alloxan-induced diabetic rats after oral administration of metformin, WSF of *Abroma augusta* L. and CMC.

Blood glucose level (mmol/L)						
Treatment groups	0 hr	2 hrs	4 hrs	8 hrs	12 hrs	24 hrs
NC	7.77 ± 1.49	6.46 ± 0.52	5.60 ± 1.27	6.75 ± 1.77	6.30 ± 1.84	6.90 ± 1.41
DC	30.85 ± 3.32	30.85 ± 3.75	31.45 ± 3.32	31.75 ± 3.04	32.15 ± 2.62	32.80 ± 1.70
MC	32.80 ± 0.42	25.45 ± 1.20	21.05 ± 0.35	18.80 ± 0.56	23.0 ± 0.14	25.60 ± 1.56
DMC	33.20 ± 0.84	34.15 ± 0.07^a^	34.20 ± 0^a^	25.65 ± 3.04^a^	33.0 ± 0.14^a^	33.50 ± 0.42^a^
DMF	31.85 ± 0.92	32.70 ± 0.56^ a^	30.45 ± 0.49^a^	28.30 ± 0.84^a^	32.95 ± 0.49^a^	33.35 ± 0.35^a^

The values are expressed as mean ± SD of three replicates NC: normal control; DC: diabetic control; MC: diabetic with metformin only (metformin control); DMC: diabetic with metformin and CMC; DMF: diabetic with metformin; WSF of *A. augusta* L. ^a^ *P* < 0.05 compared to DM (ANOVA followed by Dunnett's test).

## Abbreviations

CMC: Na-carboxymethylcellulose
DC: Diabetic control
DM: Diabetes mellitus
DMC: Diabetic with metformin and CMC
DMF: Diabetic with metformin and WSF of *A. augusta* L.
FC: Fasting control
FG: Fasting with glucose
FGC: Fasting with glucose and CMC
FGF: Fasting with glucose and WSF of *A. augusta* L.
MC: Diabetic with metformin only (metformin control)
NC: Normal control
WSF: Water-soluble fraction.
